# Supplementary material for: Application of a 21-Gene Recurrence Score in a Swiss Single-Center Breast Cancer Population: A Comparative Analysis of Treatment Administration before and after TAILORx
Source: Diagnostics (Basel). 2023 Dec 31;14(1):97. doi: 10.3390/diagnostics14010097 (PMC10795714; doi:10.3390/diagnostics14010097)
Supplement: Supplementary file 1 [file diagnostics-14-00097-s001.zip › diagnostics-2727097-supplementary.pdf]

Application of a 21-Gene Recurrence Score in a Swiss Single-Center Breast Cancer Population: A Comparative Analysis of Treatment Administration before and after TAILORx

Supplementary Material

Table S1. Main differences in treatment administration and therapy-associated complications, in the two populations, before (A) and after (B) publication of TAILORx study

	Cohort A	Cohort B	p-value
**Treatment administration**			
Surgery	165	161	0.352
Breast Conservative	113 (68%)	108 (67%)	
Mastectomy	47 (32%)	53 (33%)	
Postoperative Complications	21 (13%)	18 (11%)	0.764
Reconstruction	28/47 (60%)	29/53 (55%)	0.679
Radiotherapy (RT)	128 (78%)	123 (76%)	0.223
RT Complications	3 (2%)	1 (0.8%)	0.86
Dose RT	54.99 (SD 8.31)	52.35 (SD 7.88)	0.417
Adjuvant CHT	31 (19%)	35 (22%)	0.763
CHT in patients ≤ 50 years old	7/40 (17.5%)	16/53 (30%)	<0.001
CHT in patients ≥ 51 years old	24/125 (19%)	20/108 (19%)	0.664
CHT in patients with RS 11-20	5 (6%)	9 (13%)	0.449
CHT in patients with RS 21-25	9 (35%)	4 (17%)	0.743
CHT in patients with RS 26-30	9 (75%)	7 (78%)	0.284
CHT in patients with RS >30	8 (57%)	14 (78%)	0.252
CHT Refusal	15 (9%)	6 (3.7%)	0.042
Osteo-oncologic Treatment	67 (41%)	72 (45%)	0.695
**TB Treatment Recommendation according to RS**			
ET+CHT total (%)	51 (31%)	48 (30%)	0.865
CHT-ET TB Recommendation in RS 0-10	1/31 (3%)	0/37 (0%)	

CHT-ET TB Recommendation in RS 11-25	23/106 (22%)	16/97 (17%)	
CHT-ET (RS >26)	28/28 (100%)	27/27 (100%)	
ET alone TB Recommendation	116 (70%)	117 (73%)	0.712
ET	155 (94%)	152 (94%)	0.229
ET Refusal	6 (3.8%)	9 (5.6%)	0.429

RS = Recurrence Score; ET = Endocrine Therapy; TB = Tumor Board; RT = Radiotherapy; CHT = Chemotherapy; SD = Standard Deviation. Note: Percentages were rounded.

Table S2. Administration of CHT-ET vs. ET alone before (cohort A) and after (cohort B) publication of TAILORx findings.

	CHT-ET Administration			ET Administration		
	A (%)	B (%)	Change (%)	A (%)	B (%)	Change (%)
**Total**	31 (19%)	35 (22%)	+ 3%	158 (96%)	152 (94%)	-2%
**Age ≤ 50 years old**	7 (17.5%)	16 (30%)	+12.5%	38 (95%)	53 (98%)	+3%
**Age ≥ 51 years old**	24 (19%)	20 (19%)	0%	121 (96%)	99 (93%)	-3%
**N0**	14 (15%)	16 (17%)	+3%	90 (95%)	89 (95%)	0%
**N1**	17 (25%)	19 (28%)	+3%	67 (100%)	64 (96%)	-4%
**Grade 3**	18 (29%)	20 (31%)	+2%	21 (42%)	50 (100%)	58%
**Lobular**	4 (14%)	6 (24%)	+10%	26 (90%)	23 (92%)	+2%
**Ki-67**	16 (31%)	20 (31%)	0%	50 (96%)	61 (95%)	-1%

CHT = Chemotherapy; ET = Endocrine Therapy. Note: Percentages were rounded.

Table S3. Logistic Regression model for association of patient, tumor, and treatment characteristics with chemo-endocrine therapy in cohort A (before TAILORx) and cohort B (after TAILORx)

**Cohort A**		
	**OR (95% CI)**	**p-Value**
**Age**	1.05 (1.01 – 1.11)	0.03
**Age <50/>50**	0	0.998
**N0/+**	3.32 (1.09 – 10.06)	0.034
**Grade 3 vs. G1/2**	0.4 (0.14 – 1.12)	0.082
**Ki-67% high/low (<20% vs. ≥ 20%)**	2.15 (0.74 – 6.29)	0.162
**Tumor stage (T2/3 vs. T1)**	0.97 (0.95 – 1)	0.043
**RS 18-30**	0.12 (0.03 – 0.43)	0.001
**RS>31**	0.04 (0.01 – 0.21)	<0.001
**Cohort B**		
	**OR (95% CI)**	**p-value**
**Age**	0.89 (0.84 – 0.94)	<0.001

**Age <50/>50**	0.79 (0.11 – 5.92)	0.818
**N0/+**	3.31 (1.29 – 8.53)	0.013
**Grade 3 vs. G1/2**	0.79 (0.27 – 2.26)	0.654
**Ki-67 high/low (<20% vs. ≥ 20%)**	1.1 (0.39 – 3.13)	0.853
**Tumor Size (T2/3 vs. T1)**	0.99 (0.96 – 1.01)	0.357
**RS 11-25**	0.76 (0.21 – 2.76)	0.678
**RS >26**	1.31 (0.36 – 4.76)	0.678

CHT = Chemotherapy; RS = Recurrence Score; N0/+ = nodal status; SD = Standard Deviation; OR = Odds Ratio; CI = Confidence Interval

Table S4. Chemotherapy administration in the whole study population according to patients and tumor characteristics.

	SD	p-Value	Odds Ratio (OR)	95% Confidence Interval
**Age**	0.02	0.001	0.93	0.89-0.97
**Age ≤50 />50 years old**	0.33	0.367	1.35	0.7 - 2.59
**Cohort A/Cohort B**	0.46	0.102	0.47	0.19-1.16
**Comorbidities Yes/No**	0.45	0.626	1.24	0.52-2.99
**pT**	0.35	0.086	1.81	0.92-3.56
**pN**	0.44	<0.001	4.77	2.03-11-22
**Lobular/Ductal Histology**	0.32	0.12	0.61	0.33-1.14
**Ki-67 ≤20%/>20%**	0.49	0.291	0.6	0.23-1.55
**Grade 2**	0.46	0.376	0.67	0.27-1.63
**Grade 3**	0.45	0.473	1.39	0.57 - 3.38
**Oncotype RS low (0-10)**	1.2	<0.001	0	0-0.02
**Oncotype RS intermediate (11-25)**	0.58	<0.001	0.02	0.01-0.07
**Oncotype RS high (≥26)**	1.21	<0.001	617.93	57.97 - 6587.16

CHT = Chemotherapy; RS = Recurrence Score; N0/+ = nodal status; SD = Standard Deviation; OR = Odds Ratio; CI = Confidence Interval

Table S5. Main treatment strategies and outcomes in the intermediate RS (11-25) node negative population before and after publication of TAILORx study

	Cohort A	Cohort B	p-value
**Number of patients**	66 (62%)	58 (60%)	
**Menopause**	51 (77%)	38 (66%)	0.206
**Age ≤ 50 years old**	15 (23%)	19 (33%)	0.338

**Age ≥ 51 years old**	51 (77%)	39 (67%)	0.205
**Surgery**			
**Breast conservative**	50 (76%)	37 (64%)	0.114
**No surgical reconstruction**	28 (42%)	29 (50%)	0.69
**CHT**	6 (9%)	3 (5%)	0.49
**CHT refusal**	5 (45%)	0 (0%)	
**CHT in premenopausal women with RS >16**	1 (16%)	2 (67%)	
**RT**	46 (70%)	40 (69%)	0.969
**RT refusal**	4 (8%)	2 (5%)	0.904
**Mean RT Dose**	54.14 (SD 6.8, IQR 9.52)	50.31 (SD 7.16, IQR 2.8)	0.021
**ET**	62 (94%)	52 (90%)	0.326
**ET refusal**	3 (5%)	6 (10%)	
**Osteo-oncologic treatment**	29 (44%)	23 (40%)	0.91
**Relapse**	4 (6%)	2 (3%)	0.516
**Death**	2 (3%)	0 (0%)	
**Mean Follow-up (months)**	60.52 (SD 22.03, IQR 27)<br>Range: [4-108]<br>Median: 61	18.5 (SD 11.47, IQR 16.5)<br>Range: [1-41]<br>Median: 17	<0.001
**TB Implementation**	50 (76%)	48 (83%)	0.919

CHT = Chemotherapy; RS = Recurrence Score; RT = Radiotherapy; ET = Endocrine Therapy; TB = Tumor Board; SD = Standard Deviation. Note: Percentages were rounded.

Table S6. Association of chemotherapy administration according to age and RS

Cohort A	SD	p-Value	OR	95% CI
Age ≤ 50 years old	0.11	0.835	1.02	0.83 – 1.26
RS for Patients ≤ 50 years old	0.13	0.009	0.75	0.56 – 0.92
Age ≥ 51 years old	0.05	0.006	1.13	1.04 – 1.24
RS for Patients ≥ 51 years old	0.04	<0.001	0.81	0.74 – 0.88
Cohort B	SD	p-Value	OR	95% CI
Age ≤ 50 years old	0.07	0.552	0.96	0.84 – 1.1
RS for Patients ≤ 50 years old	0.08	0.001	1.29	1.11 – 1.49
Age ≥ 51 years old	0.05	<0.001	0.81	0.74 – 0.89
RS for Patients ≥ 51 years old	0.04	0.132	1.06	0.98 – 1.14

CHT = Chemotherapy; RS = Recurrence Score; SD = Standard Deviation; OR = Odds Ratio; CI = Confidence Interval
